# Global Pain Trends and Disparities: A Longitudinal Study of Adults 50+ Across Europe, Asia, and the Americas

**DOI:** 10.1093/geroni/igaf122.1906

**Published:** 2025-12-31

**Authors:** Esteban Calvo, Jose Medina, Antonia Diaz-Valdes

**Affiliations:** Universidad Mayor, Santiago, Region Metropolitana, Chile; Universidad Mayor, Santiago, Region Metropolitana, Chile; Universidad Mayor, Santiago, Region Metropolitana, Chile

## Abstract

Pain is a prevalent condition among older adults, significantly impacting quality of life and healthcare systems. Understanding cross-national pain trends and disparities can inform targeted interventions and policies. This study examines long-term pain trends and social disparities in 22 countries across three continents. Using harmonized longitudinal data from six surveys, we analyzed 212,904 adults aged 50+ observed repeatedly between 1998 and 2018. Pain prevalence trends from 2006 to 2016 were estimated, adjusting for survey differences. Disparities by sex, education, and age were assessed using logistic regression models. The unadjusted pain prevalence across all countries and years was 43.2%. Instrument-adjusted prevalence varied from 26.7% (Netherlands, 2006) to 59.2% (France, 2016). Pain prevalence increased in 15 countries (mean annual increase: 1.21 percentage points; p < 0.05) and decreased only in China (-0.66 percentage points per year; p < 0.05). Pain was higher among women, less educated individuals, and older adults, with disparities varying significantly across countries and subtly over time. South Korea exhibited the largest disparities, while Denmark had the smallest. Some countries (e.g., Spain, Italy) showed widening disparities, whereas others (e.g., Denmark, Sweden) exhibited stable or decreasing trends. The substantial cross-national variation in pain prevalence and disparities suggests that pain can be mitigated through policy and intervention efforts. Identifying high-disparity regions enables targeted strategies, while lessons from low-disparity regions may inform best practices.

